# RE: Routine clozapine assay monitoring to improve the management of treatment-resistant schizophrenia

**DOI:** 10.1192/bjb.2022.90

**Published:** 2023-04

**Authors:** Sumeet Gupta, Liyana Nur Mohamad

**Affiliations:** Consultant Psychiatrist, Tees, Esk and Wear Valleys NHS Foundation Trust, UK. Email: sumeet.gupta@nhs.net; Senior Registrar, Tees, Esk and Wear Valleys NHS Foundation Trust, UK

13 November 2022

*Routine clozapine assay monitoring should start during initial titration of clozapine dosages*

Kitchen and colleagues have published a very clinically relevant paper highlighting the importance of routine clozapine assays.^1^ We would support their conclusion of increased use of routine monitoring using clozapine assays. However, it is difficult to fathom why routine therapeutic drug monitoring (TDM) is still not considered justifiable in clinical practice. Clozapine fulfils the criteria for TDM as it has a narrow therapeutic index, substantial inter-individual variations in daily dose and plasma concentration relationship, and complex metabolism. We suggest that TDM should start during the initial titration to determine the target dose, rather than being restricted to annual monitoring.

The *British National Formulary* mentions that the usual dose of clozapine is between 200 and 450 mg, and the maximum is 900 mg per day. The Summary of Product Characteristics of clozapine further suggests that once patients have achieved maximum therapeutic benefit, many can be maintained effectively on lower doses. Owing to the significant inter-individual variations in bioavailability (up to 50 times), complex metabolism and wide range of recommended oral dosages, in clinical practice the dosage of clozapine is usually guided by nomograms. These nomograms were developed many years ago to predict plasma levels of clozapine taking into account only two covariates, gender and smoking status. These nomograms’ predictive values are associated with wide confidence intervals.^2^ Hence, it is not surprising that the authors found significant variations in plasma clozapine levels (<0.1 to >1 mg/L). We are assuming that most patients were receiving the recommended dosages of clozapine.

Clozapine is associated with many significant adverse effects, and these are the most commonly cited reasons for discontinuation during the initial phase of treatment.^3^ The most important adverse effects from the patients’ perspectives are sedation, hypersalivation and constipation, and these are probably dose-related adverse effects.^3,4^ Moreover, clozapine is the only evidence-based treatment for patients with treatment-resistant schizophrenia, and most patients will stay on it on a long-term basis; hence it is important that patients should be treated with the minimum effective dose.

The recommended plasma clozapine levels of 0.35–0.60 mg/L are for the management of active psychotic symptoms. The recommended plasma levels for maintenance treatment might be lower than the above range.^4^

The authors have highlighted concerns about high clozapine levels and their potential association with high mortality. Lower clozapine levels are also of concern, especially if a patient has partially responded to clozapine. Hence, there is now increasing support for the view that TDM should be used during the initial phase of clozapine treatment to achieve minimum therapeutic levels.^4^ Subsequently, dosages can be optimised, based on the response and side-effects burden. At present, this is difficult as clozapine assays are done by selected centres in the UK, and it can take a few days to weeks to get the results. Even then, it will be prudent to obtain plasma clozapine levels soon after the titration. In the future, point-of-care testing for clozapine might make it easier to titrate the dose to achieve the minimum therapeutic level.^5^
